# A New, Homœopathy

**Published:** 1883-03

**Authors:** 


### HOMŒOPATHIC PHYSICIAN,

A MONTHLY JOURNAL OF MEDICAL SCIENCE.

"If our school ever gives up the strict inductive method of Hahnemann, we are lost, and deserve only to be mentioned as a caricature, in the history of medicine."—CONSTANTINE HERING.

Vol. III. MARCH, 1883. No. 3.

### EDITORIAL.

A NEW HOMŒOPATHY. In our last issue we said: "Of late years the American Institute has not only ceased its good work, but is doing much to destroy the great work done by its founders." We now publish a formulated statement of its present status and creed, proving our assertion to be true. Let any one who desires to measure the Institute's degradation compare this statement with the declaration of the *founders* of the Institute (as given on page 42).

We are gratified to see that there is a growing tendency among the eclectics to *openly* repudiate all Hahnemann's teachings and to boldly announce themselves as eclectics. The opinions expressed in the following paragraphs undoubtedly give the views of the large majority of the members of the Institute. The writer clearly repudiates Homœopathy—at least as Hahnemann taught it. Now let the *name* be dropped and honesty will achieve a victory. They "seek recognition;" this will aid in securing it. Dr. Austin Flint is reported (New York *Herald*, January 30th, 1883) as saying: "Let the homœopathists do away with their organization and their name; he cared not what opinions they held."

The fine cardinal principles of new "Homœopathy:"

1. Palliative medicine is the common property of the whole medical profession and Homœopathy *must* openly assert her rights therein.
2. The American Institute holds to no exclusive dogma [law].

3. Similia similibus curantur is *only one* of several known laws of cure, available alike to Homœopathy and to *scientific medicine.*

4. The American Institute, at the Indianapolis meeting, officially *refuted* all the charges made against Homœopathy by regular medicine.

5. Homœopathy is now seeking national, state, and municipal recognition.

Do true homœopathists subscribe to these declarations? Are these the principles for which a Hahnemann, a Bœnninghausen, a Hering, or a Dunham labored?

Do honorable men, conscious of merit and rectitude, basely "seek recognition," or do they boldly demand honorable equality? *Criminals seek pardon; honest men demand justice.*

